# Thidiazuron Enhances Strawberry Shoot Multiplication by Regulating Hormone Signal Transduction Pathways

**DOI:** 10.3390/ijms26094060

**Published:** 2025-04-25

**Authors:** Fang Wang, Yali Li, Yadan Pang, Jiangtao Hu, Xinna Kang, Chun Qian

**Affiliations:** 1 Institute of Urban Agriculture, Chinese Academy of Agricultural Sciences, Chengdu 610213, China; wangfang08@caas.cn (F.W.); pangyadan@163.com (Y.P.); hujiangtao@caas.cn (J.H.); 2 Institute of Remote Sensing and Digital Agriculture, Sichuan Academy of Agricultural Sciences, Chengdu 610066, China; 3 College of Horticulture and Landscape Architecture, Southwest University, Chongqing 400712, China; qianchun1973@163.com; 4 Shijiazhuang Academy of Agriculture and Forestry Sciences, Shijiazhuang 050080, China; nkykang@163.com

**Keywords:** thidiazuron (TDZ), shoot multiplication, strawberry (*Fragaria* × *ananassa*), hormone signal transduction, transcriptome analysis

## Abstract

Tissue culture-based rapid propagation is critical for genetic improvement and virus-free production of strawberries (*Fragaria* × *ananassa*). This study evaluated the optimal concentration of thidiazuron (TDZ) for shoot multiplication and explored the underlying molecular mechanisms. Strawberry explants were treated with TDZ at concentrations of 0, 0.025, 0.05, 0.1, and 0.4 mg·L^−1^ in vitro, and growth, physiological changes, and transcriptomic profiles were analyzed after four weeks. The results identified 0.05 mg·L^−1^ TDZ as the most effective concentration for shoot proliferation, yielding a significant increase in leaf number. However, TDZ application inhibited plant height and reduced chlorophyll, carotenoid, and soluble sugar contents. Physiological analyses revealed that TDZ decreased endogenous cytokinin levels while elevating auxin concentrations. Transcriptomic analysis showed that TDZ suppressed cytokinin biosynthesis and up-regulated cytokinin oxidase expression, thereby modulating hormone homeostasis. Additionally, TDZ enhanced the cytokinin signaling pathway, which is crucial for cell division and shoot initiation, and influenced auxin, gibberellin, and brassinosteroid pathways to regulate differentiation. These findings suggest that TDZ promotes strawberry shoot multiplication primarily through hormone signal transduction, providing insights for optimizing tissue culture protocols.

## 1. Introduction

Strawberry (*Fragaria* × *ananassa*) is a herbaceous perennial plant belonging to the Rosaceae family [1]. It is one of the most popular fruits worldwide due to its beautiful appearance, rich flavor, and health benefits. Strawberry cultivation and yield have increased dramatically over the past two decades. According to the Food and Agriculture Organization (FAO), global strawberry production reached 9.57 million tons in 2022, more than double the 4.5 million tons recorded in 2000 [2].

Plant tissue culture is a set of techniques for the aseptic culture of cells, tissues, and organs under defined physical and chemical conditions [3]. This technology can be used to explore conditions that promote cell division and gene reprogramming under in vitro conditions and is considered an essential tool for basic and applied research, as well as commercial applications [4]. Plant tissue culture mainly serves five major areas nowadays, including rapid propagation of selected individuals, generation of genetically modified individuals, as a research model for fundamental aspects of plant cell physiology, preservation of endangered species, and metabolic engineering of fine chemicals [5,6].

Rapid propagation of strawberries is an essential step for genetic modification and production of virus-free plants. The speed of shoot multiplication decides the efficiency of rapid propagation, which is closely related to the extensive germination of axillary buds [7]. Cytokinins are indispensable for the propagation of plants in tissue culture, and they are in concert with the plant hormone auxin to stimulate cell division. It has been reported that cytokinin and auxin coordinate the dormancy and outgrowth of axillary bud in strawberry [8]. The cytokinins used for tissue culture include benzyl adenine (BA), kinetin, and zeatin. BA is a commonly used cytokinin for strawberry multiplication in vitro [9]. However, the efficiency of BA on strawberry multiplication remains limited.

Axillary bud germination is a complex process. The cytokinin-induced axillary bud germination mechanism has been widely studied in recent years. The expression of isopentenyltransferase (*IPT*), a key gene of cytokinin synthesis, was positively correlated with axillary bud initiation [10,11,12], while the expression of cytokinin oxidase/dehydrogenase (*CKX*) was negatively correlated [13]. A current model for cytokinin signal transduction has been proposed in recent years. The ARABIDOPSIS HIS KINASEs (AHKs) are cytokinin receptors, and ARABIDOPSIS HIS PHOSPHO TRANSFER PROTEINS (AHPs) transfer the signal from AHKs to ARABIDOPSIS RESPONSE REGULATORS (ARRs), ARRs is located in the nucleus. It stimulates the transcription of cytokinin-responsive genes [14,15]. The ARRs can be grouped into type-A ARRs and type-B ARRs according to the C-terminal domain structure. Generally, type-B ARRs are considered positive regulators of the cytokinin signal transduction pathway, while type-A ARRs are the typical negative regulators [16]. Genetic studies in *Arabidopsis* and other species have found that axillary bud initiation is regulated by several crucial transcription factors—for instance, WUSCHEL (WUS), Cup-Shaped Cotyledon (CUC), and Shoot Meristemless (STM). Type-B ARRs can directly bind to the *WUS* promoter to activate its de novo expression and promote axillary bud differentiation [17]. The activity of *CUC* and *STM* is also partly dependent on the cytokinin pathway [18,19]. Moreover, a recent study demonstrated that cytokinin affects the expression of auxin efflux transporter *PIN3*, *PIN4*, and *PIN7* to promote axillary bud differentiation in *arr1* mutant [20]. These results suggest that there may be several signaling pathways for cytokinin to regulate axillary bud differentiation.

Thidiazuron (TDZ) belongs to bis-substituted urea derivatives, which showed extreme cytokinin activity [21]. TDZ is a potent regulator that can affect multiple morphogenetic processes, including axillary shoot induction, adventitious shoot regeneration, and somatic embryogenesis in many plant species. Researchers found that TDZ induces as many or more shoots as adenine-type cytokinins (BA, kinetin, and zeatin) for most species [22]. Previous research has also shown that TDZ is more effective than adenine-type cytokinins for axillary bud differentiation in strawberries [23]. This study aimed to determine the proper concentration of TDZ for shoot multiplication in strawberries and to explore the regulation pathway of TDZ on the proliferation and differentiation of strawberry axillary buds. This study is beneficial for accelerating strawberry genetic research and the production of virus-free strawberry plants.

## 2. Results

### 2.1. Plant Growth and Multiplication

**Figure 1:**
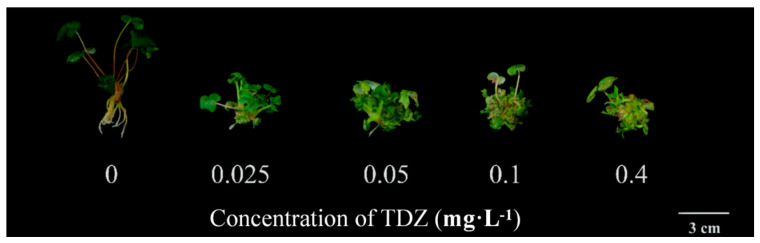
Strawberry plants affected by TDZ after 4 weeks of cultivation.

**Figure 2:**
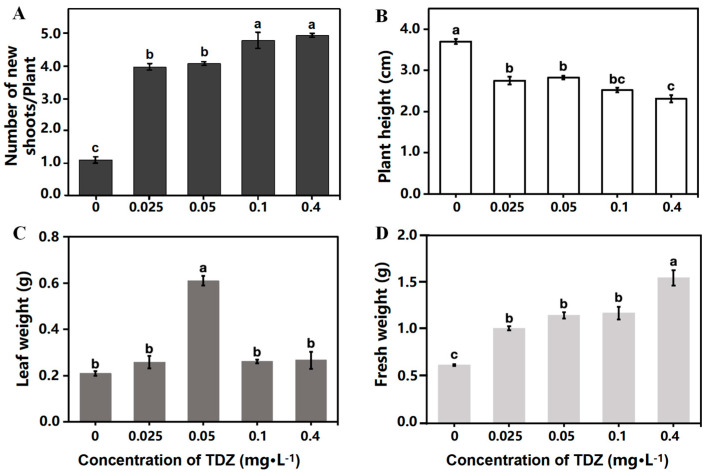
Strawberry growth affected by different concentrations of TDZ. (**A**): Number of new shoots per plant; (**B**): Plant height; (**C**): Leaf weight; (**D**): Fresh weight. Lowercase letters indicate significant differences calculated by Duncan’s multiple range test at *p* ≤ 0.05. Vertical bars indicate the standard error (*n* = 3).

Strawberry growth parameters were collected after 4 weeks of cultivation. The plant growth was significantly different among treatments (Figure 1). More shoots were produced (Figure 2A), while the height of new shoots was decreased with the increased concentration of TDZ (Figure 2B). The highest shoot number (4.93/plant) was observed at 0.4 mg·L^−1^ TDZ. Number of shoots in 0.025 mg·L^−1^ (3.96/plant) and 0.05 mg·L^−1^ (4.07/plant) TDZ treatments showed no significant difference. The highest leaf weight was achieved when TDZ concentration was 0.05 mg·L^−1^ (Figure 2C), which indicates that 0.05 mg·L^−1^ TDZ was the best concentration for shoot multiplication in strawberries. Higher concentrations of TDZ induced more callus, which caused higher fresh weight compared with other concentrations (Figure 2D).

### 2.2. Contents of Chlorophyll and Carotenoid

**Figure 3:**
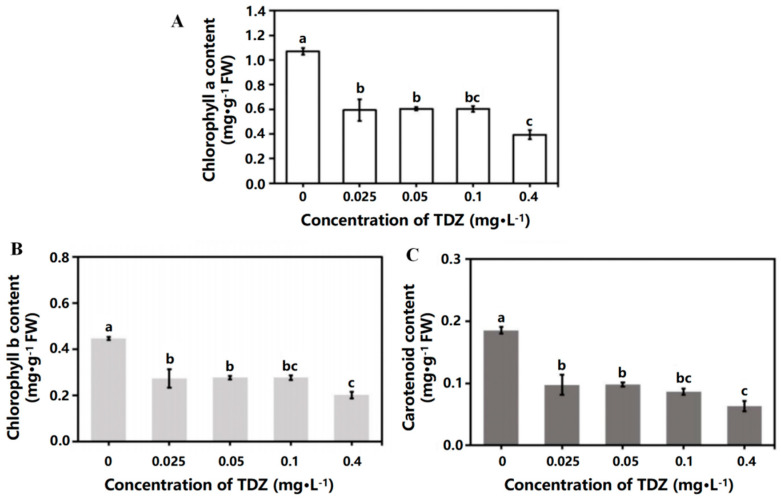
Chlorophyll and carotenoid contents affected by TDZ. (**A**): Chlorophyll a content; (**B**) Chlorophyll b content; (**C**): Carotenoid content. Lowercase letters indicate significant differences calculated by Duncan’s multiple range test at *p* ≤ 0.05. Vertical bars indicate the standard error (*n* = 3).

TDZ application significantly reduced the contents of chlorophyll a, chlorophyll b, and carotenoids in leaves (Figure 3A–C). There was no significant difference in chlorophyll and carotenoid contents when TDZ concentrations were 0.025, 0.05, and 0.1 mg·L^−1^. The contents of chlorophyll a, chlorophyll b, and carotenoids were significantly lower when TDZ concentration was 0.4 mg·L^−1^ (*p* < 0.05).

### 2.3. Contents of Soluble Sugar and Protein

**Figure 4:**
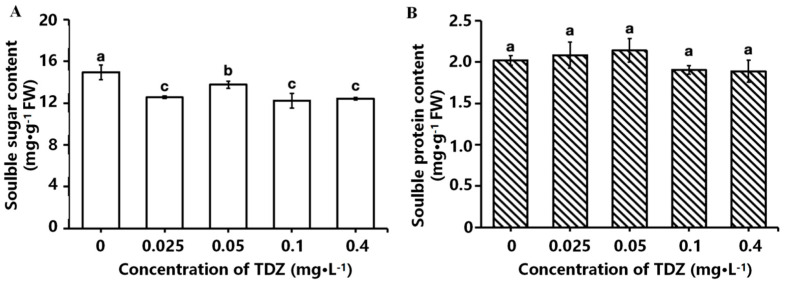
Effects of different TDZ concentrations on the contents of soluble sugar (**A**) and protein (**B**) in strawberries. Lowercase letters indicate significant differences calculated by Duncan’s multiple range test at *p* ≤ 0.05. Vertical bars indicate the standard error (*n* = 3).

The effects of different TDZ concentrations on the contents of soluble sugar and soluble protein are shown in Figure 4A,B. Soluble sugar content in all TDZ-treated plants was significantly decreased (*p* < 0.05) compared to the control. However, plants treated with 0.05 mg·L^−1^ TDZ had the most minor reduction and was higher than the other three TDZ treatments. There was no significant difference in soluble protein content among different TDZ treatments.

### 2.4. Contents of Cytokinin and Auxin Contents

**Figure 5:**
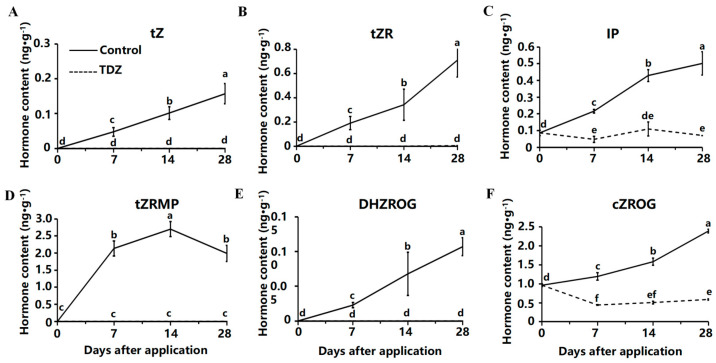
Contents of cytokinin affected by TDZ during plant growth. (**A**): trans-zeatin, tZ; (**B**): trans-zeatin riboside, tZR; (**C**): N6-isopentenyladenine, IP; (**D**): 9-ribosyl-trans-zeatin 5′-monophosphate, tZRMP; (**E**): dihydrozeatin-O-glucoside riboside, DHZROG; (**F**): cis-zeatin-O-glucoside riboside, cZROG. Error bars represent the standard errors of the three replications (*n* = 3). Lowercase letters indicate the significant difference according to Duncan’s multiple range test at range test at a 0.05 level.

The total cytokinin content in strawberries treated with TDZ was significantly lower than in the control. Six CK showed an increasing trend in the medium without TDZ, including trans-zeatin (tZ) (Figure 5A), trans-zeatin riboside (tZR) (Figure 5B), N6-isopentenyladenine (IP) (Figure 5C), 9-ribosyl-trans-zeatin 5′-monophosphate (tZRMP) (Figure 5D), dihydrozeatin-O-glucoside riboside (DHZROG) (Figure 5E), cis-zeatin-O-glucoside riboside (cZROG) (Figure 5F). Less endogenous CK was detected in TDZ treatment, and although the content of tZ, tZR, tZRMP, and DHZROG were increased in control, they were hardly detected in TDZ treatment.

**Figure 6:**
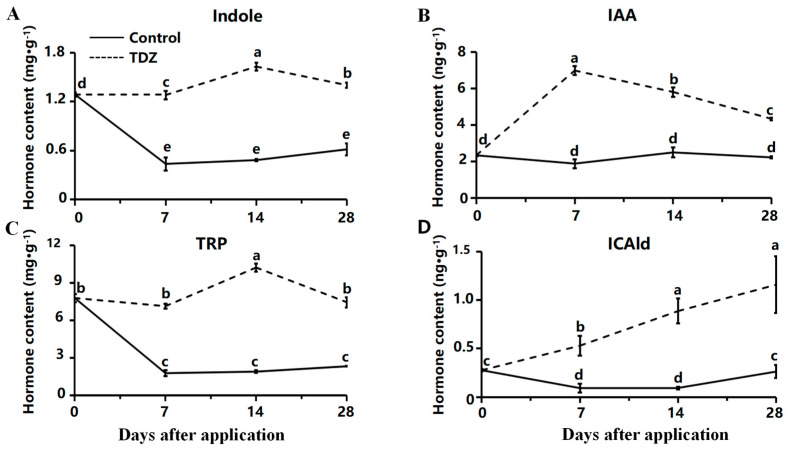
Contents of auxin affected by TDZ during plant growth. (**A**): Indole; (**B**): Indole-3-acetic acid, IAA; (**C**): L-tryptophan, TRP; (**D**): Indole-3-carboxaldehyde, ICAld. Error bars represent the standard errors of the three replications (*n* = 3). Lowercase letters indicate the significant difference according to Duncan’s multiple range test at range test at a 0.05 level.

The total contents of auxin were increased after the TDZ application compared with the control. Contents of indole and L-tryptophan (TRP) were maintained at a high level after TDZ application (Figure 6A,C). In contrast, the level of those two auxins was significantly reduced during cultivation in control. The content of IAA was increased at the beginning and then decreased after the TDZ application (Figure 6B). Content of Indole-3-carboxaldehyde (ICAld) was increased linearly with TDZ (Figure 6D).

### 2.5. Transcriptome Analysis

**Figure 7:**
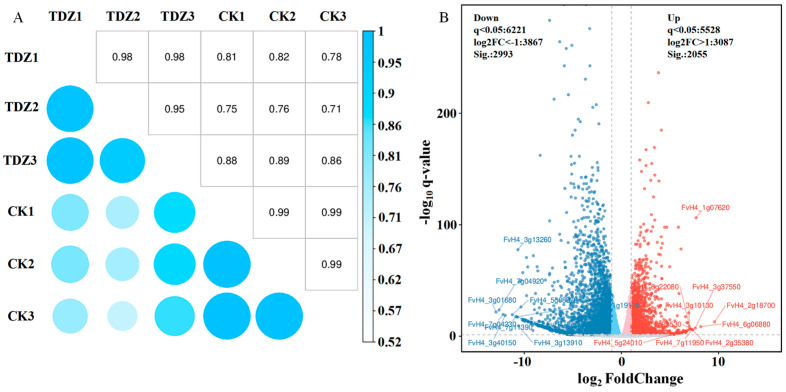
Correlation coefficient analysis (**A**) and volcano map (**B**) of different expressed genes between TDZ treated plants and control.

Six cDNA libraries were established using RNA from strawberries treated with or without TDZ. After data filtering, 45.16–48.75 M clean reads for each library were obtained. The average GC content was 47.00%, and the average value of base quality Q30 was 94.63% (Table S3). The ratio of clean reads mapped to the reference genome ranged from 94.06–95.99%. The correlation coefficient between samples reflects data from TDZ and control repeats, which have high consistency (Figure 7A). The correlation coefficient between TDZ and control was low (0.71–0.89), indicating that gene expression was quite different between TDZ and control. The volcano map revealed that 5048 genes were differently expressed between TDZ and control (Figure 7B). Two thousand fifty-five genes were significantly up-regulated, and 2993 were significantly down-regulated after TDZ application.

#### 2.5.1. GO Analysis

**Figure 8:**
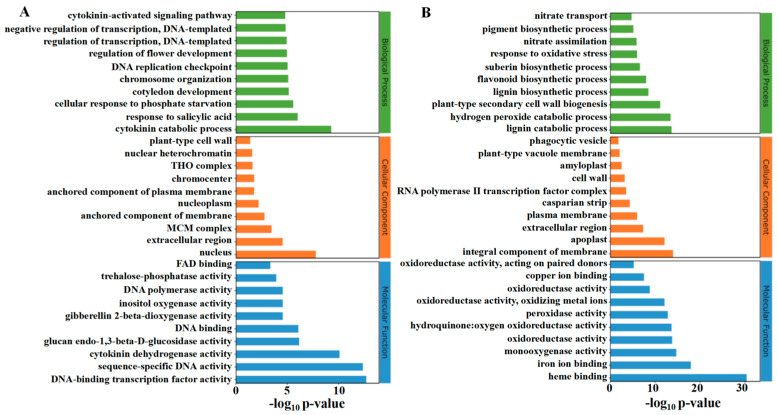
GO enrichment analysis of up (**A**) and down (**B**) -regulated differentially expressed genes.

The classification of the top thirty up-regulated and down-regulated functions in GO enrichment analysis is shown in Figure 8A,B. TDZ up-regulated biological processes include the cytokinin catabolic process, response to salicylic acid, cellular response to phosphate starvation, cytokinin-activated signaling pathway, and DNA replication-related process. The top three up-regulated cellular components by TDZ were the nucleus, extracellular region, and MCM complex. The up-regulated molecular function by TDZ includes several DNA-binding activities, cytokinin dehydrogenase activity, glucan endo-1,3-beta-D-glucosidase activity, and trehalose-phosphatase activity. The main down-regulated biological processes caused by TDZ include lignin biosynthetic and catabolic processes, hydrogen peroxide catabolic process, plant-type secondary cell wall biogenesis, suberin biosynthetic process, nitrate transport and assimilation processes, flavonoid biosynthetic process. Cellular components—including the integral components—of the membrane, apoplast, and extracellular region were significantly down-regulated by TDZ. Molecular functions, including heme binding, iron ion binding, and monooxygenase activity, were also significantly down-regulated in TDZ treatment.

#### 2.5.2. KEGG Analysis

**Figure 9:**
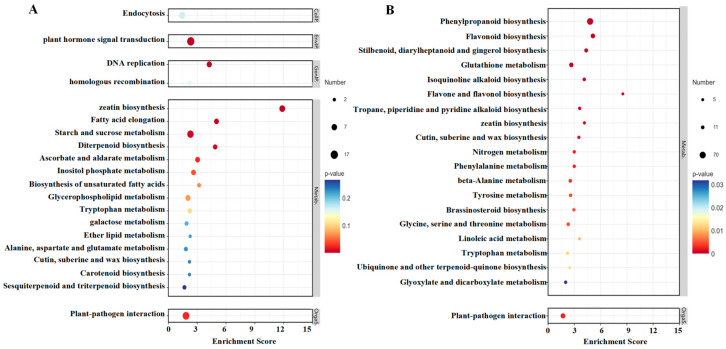
KEGG enrichment analysis of up-regulated (**A**) and down-regulated (**B**) pathways in strawberries treated with TDZ.

The results of the KEGG enrichment analysis of up-regulated and down-regulated pathways in strawberries treated with TDZ are shown in Figure 9A,B. The most significant pathway affected by TDZ was zeatin biosynthesis since it was significantly up-regulated and down-regulated to some extent. DNA replication, fatty acid elongation, diterpenoid biosynthesis, starch and sucrose metabolism, and plant hormone signal transduction were significantly up-regulated after TDZ application. Several phenylpropanoid pathways, such as phenylpropanoid biosynthesis, flavonoid biosynthesis, flavone and flavonol biosynthesis, and phenylalanine metabolism, were significantly reduced by TDZ.

#### 2.5.3. Zeatin Synthesis

**Figure 10:**
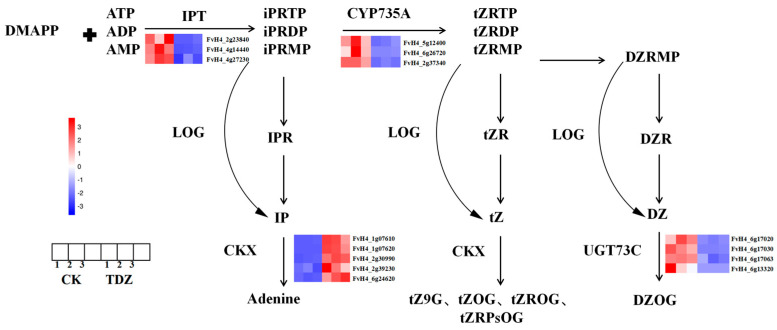
Zeatin biosynthesis pathway as affected by TDZ. Abbreviations of cytokinin: IPR: N6-(2-isopentenyl)adenine 9-riboside; IP: N6-(2-isopentenyl)adenine; iPRMP: N6-(2-isopentenyl)adenine 9-riboside-5′-monophosphate; tZ: trans-zeatin; tZR: trans-zeatin 9-riboside; tZOG: trans-zeatin O-glucoside; tZROG: trans-zeatin 9-riboside O-glucoside; tZRMP: trans-zeatin 9-riboside-5′-monophosphate; DZRMP: dihydrozeatin 9-riboside-5′-monophosphate; DZR: dihydrozeatin 9-riboside; DZ: dihydrozeatin; DZOG: dihydrozeatin O-glucoside. Abbreviations of enzymes: IPT: isopentenyltransferase; LOG: LONELY GUY; CKX: cytokinin oxidase/dehydrogenase.

A total of 18 DEGs are found in the zeatin pathway, as shown in Figure 10. Three *IPT* (FvH4_2g23840, FvH4_4g14440, FvH4_4g27230) and three *CYP735A* (FvH4_6g45180, FvH4_6g26720, FvH4_2g37340) that are very important for zeatin biosynthesis were significantly decreased after plants treated with TDZ. The expression of five cytokinin degradation genes *CKX* (FvH4_1g07610, FvH4_1g07620, FvH4_2g30990, FvH4_2g39230, FvH4_6g24620), as well as three *CKX-like* genes (FvH4_3g03260, FvH4_3g04610, FvH4_7g02150) were significantly increased by TDZ application. Another four *UGT73C* (FvH4_6g17020, FvH4_6g17030, FvH4_6g17063, FvH4_6g13320), which could convert DZ to DZOG were down-regulated by TDZ.

#### 2.5.4. Plant Hormone Signal Transduction

**Figure 11:**
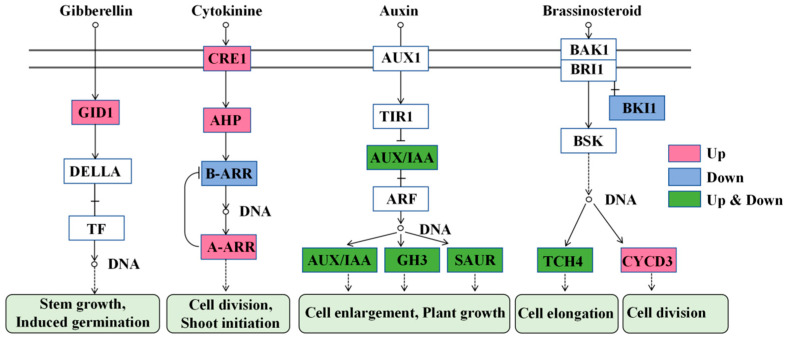
Plant hormone signal transduction affected by TDZ. GID1: Gibberellin insensitive dwarf 1; TF: Transcription factor; CRE1:Cytokinin response 1; AHP: Cytokinin response 1; ARR: Arabidopsis response regulators; AUX1: Auxin resistant 1; TIR1: Transport inhibitor response 1; ARF: Auxin response factors; GH3: Gretchen Hagen3; SAUR: Small auxin up-regulated RNA; BRI1: Brassinosteroid insensitive 1; BAK1: BRI1-associated kinase1; BKI1: BRI1 kinase inhibitor 1; BSK: Brassinosteroid signaling kinases; TCH4: TOUCH4; CYCD3:cyclin D3.

The cytokinin signal pathway was the most significant pathway affected by TDZ (Figure 11). This pathway is essential for cell division and shoot initiation. Two cytokinin receptor *Cytokinin response 1* (*CRE1*, FvH4_3g03620, FvH4_4g33130), one *histidine phosphotransfer protein* (*AHP*, FvH4_3g01870), and four type-A ARR (FvH4_2g27180, FvH4_4g35230, FvH4_5g16240, FvH4_7g25970) were significantly up-regulated, while one type-B ARR (FvH4_4g24870) was down-regulated by TDZ. The auxin signal pathway was much more complicated after TDZ application. TDZ increased the expression of two *AUX/IAA* (FvH4_1g02700, FvH4_1g04240), five *Gretchen Hagen3* (*GH3*, FvH4_1g16980, FvH4_2g04750, FvH4_2g25330, FvH4_3g04010, FvH4_3g21460), and one *small auxin up-regulated RNA* (*SAUR*, FvH4_2g38720). However, it also down-regulated three *AUX/IAA* (FvH4_4g02260, FvH4_4g02280, FvH4_6g02870), two *GH3* (FvH4_2g24250, FvH4_4g22430), and nine *SAUR* (FvH4_2g10760, FvH4_2g10850, FvH4_2g10870, FvH4_3g13730, FvH4_5g08780, FvH4_6g19170, FvH4_6g36860, FvH4_7g11280, FvH4_7g32600). TDZ increased the level of gibberellin receptor *Gibberellin insensitive dwarf 1* (*GID1*, FvH4_3g11720), which is beneficial for stem growth and induces axillary bud germination. The brassinosteroid signal pathway was also affected by TDZ, with three cyclin genes *CYCD3* (FvH4_1g16680, FvH4_2g05460, FvH4_2g33760) significantly increased for cell division, and TDZ repressed the expression of *brassinosteroid insensitive 1 kinase inhibitor 1* (*BKI1*, FvH4_2g34560). The expression of two *TOUCH4* (*TCH4*, FvH4_3g00840, FvH4_4g09160) genes were increased, while one *TCH4* (FvH4_4g06700) gene was decreased after TDZ application.

### 2.6. Expression Profile Validation

**Figure 12:**
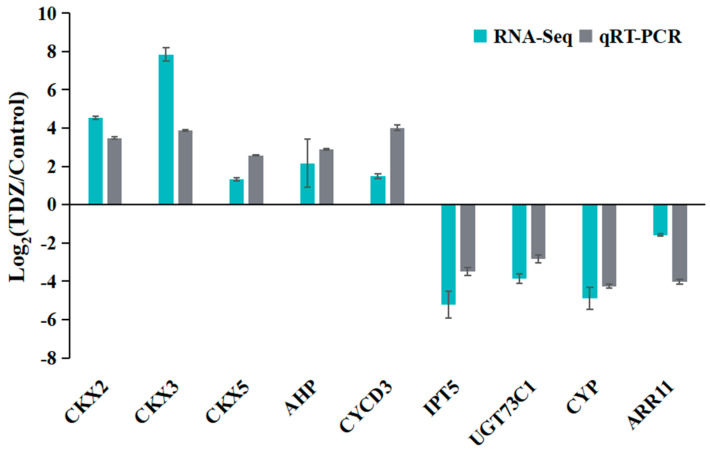
Validation of differentially expressed genes by qRT-PCR.

To validate the accuracy and reliability of the RNA-seq, a total of nine DEGs, including one type-A ARR (*ARR11*), were selected for qRT-PCR analysis. As shown in Figure 12, relative expression changes after TDZ treatments were represented using Log_2_ (TDZ/Control). All selected genes detected by qRT-PCR showed expression changes that were consistent with RNA-Seq results, suggesting that the sequencing data were credible.

## 3. Discussion

TDZ significantly enhanced shoot multiplication in strawberries, consistent with its effects in other plant species, such as red pepper (*Capsicum*) [24], *Rauvolfia tetraphylla* [25], and *Cassia sophera* [26]. However, the mechanism by which TDZ regulates the formation of a large number of shoots has rarely been reported. Cytokinin synthesis, degradation, and signal transduction were significantly affected by TDZ in this study. TDZ inhibited the synthesis of endogenous cytokinin by down-regulating the expression of several *IPT* and *CYP735A* and caused lower concentrations of endogenous cytokinin such as IP, tZRMP, tZR, tZ, and DZOG than control. On the contrary, TDZ significantly promoted the expression of *CKX*. These results indicate that TDZ-treated plants sense the accumulation of exogenous cytokinin and maintain cell homeostasis by inhibiting the synthesis of endogenous cytokinin and promoting cytokinin degradation. TDZ promoted axillary bud differentiation mainly by enhancing cytokinin signal transduction. The expression level of CRE1, AHP, and type-A ARRs was significantly increased after TDZ application. Researchers found that cytokinin receptor CRE1 can be activated by TDZ [27]. CRE1 was identical to Arabidopsis histidine kinase 4 (AHK4) [28]. It is a sensor histidine kinase that autophosphorylates after the activation by cytokinin. Although type-A ARRs are generally recognized as suppressors of cytokinin signaling, TDZ significantly increased the expression of several type-A ARRs in this study. Muller et al. [10] found that transcript abundance of a clade of type-A ARRs increased after cytokinin supply in *Arabidopsis thaliana*, suggesting that type-A ARRs are necessary for cytokinin-mediated axillary bud germination. Moreover, the reduced transcriptional abundance of type-B ARR in this study may be caused by the feedback regulation of type-A ARR.

Auxin collaborates with cytokinin to regulate bud outgrowth, which is well known [29,30,31]. The content of IAA and its synthetic precursors (indole and TRP) increased after TDZ supply in this study, suggesting that TDZ improved auxin synthesis. Jones et al. found that cytokinin directly induces auxin biosynthesis in *Arabidopsis*, and this process involves a homeostatic feedback loop regulated via auxin and cytokinin signal transduction [32]. The expression of auxin signaling genes, including *Aux/IAA*, *GH3*, and *SAUR,* was significantly affected by TDZ. The Aux/IAA, GH3, and SAUR proteins are well-known as the early auxin response proteins, and Aux/IAA acts as a hub factor that adjusts auxin signals at different auxin levels [33]. The GH3 encodes auxin amide synthetase and regulates plant growth and stresses by regulating auxin homeostasis [34,35]. The complex expression of these two proteins suggested that they both worked to regulate auxin homeostasis disrupted by TDZ. SAUR was essential for cell elongation in many plants [36]. The dwarf phenotype of plants treated with TDZ may regulated through the nine down-regulated SAURs. The auxin, GA, and brassinosteroids modulate cell expansion and proliferation and are well-known for overlapping activities in physiological assays [37,38]. It has been identified that auxin can stimulate the biosynthesis of GA and brassinosteroids [39,40]. The raised expression of GID1, TCH4, and CYCD3 in TDZ-treated plants in this study indicated that auxin improved GA and brassinosteroid pathways. However, the crosstalk between auxin and those two hormones for regulating axillary bud initiation and outgrowth is complicated and needs further research.

Chlorophyll and carotenoids are essential molecules in higher plants that determine photosynthetic efficiency. TDZ significantly reduced chlorophyll and carotenoid contents in this study. Previous work also showed that TDZ application decreased chlorophyll content and caused obvious chlorosis [23]. Transcriptome analysis revealed that the lignin biosynthetic and catabolic processes and the suberin biosynthetic processes were significantly down-regulated by TDZ. Lignin is synthesized mainly in vascular plants and is part of the nutrient transport system [41]. Suberin is a lipophilic extracellular barrier located on the inner side of the primary cell wall. It functions to control fluxes of water and solutes [42]. The reduced synthesis of these two compounds in TDZ-treated plants caused less assimilation of nutrients. Since nitrate transport and assimilation processes, iron ion binding and heme binding were significantly decreased after TDZ application, suggesting that absorption of nitrogen and iron was significantly hindered, resulting in the reduction in chlorophyll and carotenoid synthesis.

Soluble sugar in plants contains most monosaccharides and oligosaccharides, such as glucose, fructose, sucrose, and trehalose. TDZ significantly reduced soluble sugar content. This is mainly due to the reduced chlorophyll and carotenoid content, which causes inefficient photosynthesis. Plants treated with 0.05 mg·L^−1^ TDZ possessed more soluble sugar than other TDZ-treated plants because more leaves in plants treated with 0.05 mg·L^−1^ TDZ produced more photosynthetic product. Although TDZ reduced photosynthetic efficiency, starch and sucrose metabolism was enhanced. The trehalose phosphatase activity and glucan endo-1,3-beta-D-glucosidase activity also significantly increased in TDZ treatment. Trehalose is an intermediate product of starch and sucrose metabolism. Trehalose and other sugars are crucial in plant growth and development [43]. They can interact with other signaling pathways to adjust downstream responses and then regulate cell proliferation, expansion, and differentiation [44,45]. These results indicated that sugar may play a vital role in axillary bud differentiation in strawberries.

The phenylpropanoid pathway is responsible for synthesizing more than 8000 metabolites, contributing to plant growth and environmental responses. Flavonoid metabolism is an important branch of phenylpropanoid metabolism, containing over 6000 compounds. Phenylpropanoid metabolism is strongly related to diverse phytohormone signal pathways, such as auxin and GA [46]. Shi et al. [47] revealed that the decrease in phenylpropanoid and flavonoid biosynthesis is an essential factor in improving auxin transport and promoting axillary bud growth. The phenylpropanoid biosynthesis, flavonoid biosynthesis, flavone and flavonol biosynthesis, and phenylalanine metabolism were significantly down-regulated by TDZ in this study, suggesting that the reduction of phenylpropanoid pathway may play a role in promoting the growth of strawberry axillary buds by enhancing auxin transport.

This work provides a foundation for optimizing TDZ-mediated strawberry propagation. However, the study only focused on the cultivar ‘Benihoppe’. Testing TDZ on multiple strawberry cultivars and wild relatives to assess genotype-specific responses will help to better understand the proliferation effect of TDZ on strawberries. Long-term effects of TDZ on root development, flowering, and fruit quality should also be evaluated in the future.

## 4. Materials and Methods

### 4.1. Materials Preparation

Health and clean runner tips of strawberry ‘Benihoppe’ were collected from a strawberry farm located in the south of Chengdu, China. The 2–4 cm long runner tips were washed with detergent and then soaked in running water for 2 h. Thereafter, the runner tips were washed in 75% (*v*/*v*) ethanol solution for 60 s on a clean bench, followed by three rinses with distilled water. Runner tips were then soaked in 2% (*v*/*v*) sodium hypochlorite for 5 min and subsequently washed with sterile water six times. A scalpel is then used to cut the tip of the stem to 0.5 cm long and put the tips on Murashige and Skoog (MS) medium (Murashige and Skoog medium including vitamins, Duchefa Biochemie, Haarlem, The Netherlands) supplemented with 30 g·L^−1^ sucrose, 7 g·L^−1^ agar, 0.5 mg·L^−1^ 6-BA and 0.2 mg·L^−1^ NAA. Shoot tips were transferred to a culture room at 25 °C/22 °C (day/night) with 70% humidity, a 50 μmol·m^−2^·s^−1^ photosynthetic photon flux density (PPFD) white light emitting diodes (LEDs) light for 16 h photoperiod was supplied. Clustered shoots were formed 2 months later.

### 4.2. TDZ Supplement and Plant Growth Measurement

Strawberry shoots with three leaves were transferred to MS medium supplemented with 0, 0.025, 0.05, 0.1, and 0.4 mg·L^−1^ TDZ and 0.1 mg·L^−1^ NAA. Each medium contained three shoots with three replications for each treatment. All the mediums were put into a culture room with a temperature of 25 °C/22 °C (day/night) and with 50 μmol·m^−2^·s^−1^ PPFD red and blue (7:3) LEDs light for 16 h photoperiod. The number of new shoots, plant height, fresh weight of shoots, and leave weight were measured 4 weeks later. Samples of shoots were collected and stored in the −80 °C refrigerator immediately.

### 4.3. Determination of Chlorophyll Contents

The contents of chlorophyll a, chlorophyll b, and carotenoid were determined according to Lin et al. [48]. Briefly, 0.2 g of fresh leaves were weighed and submerged in 80% acetone solution. After reactions in the dark for 12 h until the leaves turned completely white, the mixture solution was used for chlorophyll content measurement. The absorbance value of liquid supernatant was measured at 663, 645, and 470 nm using a spectrophotometer (Multiskan GO, Thermo Co., Ltd., Waltham, MA, USA). Contents of chlorophyll a and b were determined using the following formulae:

$$\mathrm{Chlorophyll}\;a=\frac{\left(11.75\;\times \;\mathrm{OD}\;\mathrm{at}\;663\;\mathrm{nm}-2.35\;\times \;\mathrm{OD}\;\mathrm{at}\;645\;\mathrm{nm}\right)\;\times \;V*}{\mathrm{Sample}\;\mathrm{fresh}\;\mathrm{weight}}$$

$$\mathrm{Chlorophyll}\;b=\frac{\left(27.05\;\times \;\mathrm{OD}\;\mathrm{at}\;645\;\mathrm{nm}-11.21\;\times \;\mathrm{OD}\;\mathrm{at}\;663\;\mathrm{nm}\right)\;\times \;V*}{\mathrm{Sample}\;\mathrm{fresh}\;\mathrm{weight}}$$

$$\mathrm{carotenoid}=\frac{\left(1000\;\times \;\mathrm{OD}\;\mathrm{at}\;470\;\mathrm{nm}\;-\;2.27\;\times \;\mathrm{Chlorophyll}\;a-81.4\right)\;\times \mathrm{Chlorophyll}\;b}{227}$$

^∗^ V, the volume of the extraction solution.

### 4.4. Determination of Soluble Sugar and Protein Contents

The contents of soluble sugar and protein were determined according to Lin et al. [49]. To estimate the soluble sugar contents, 0.5 g leaf samples were homogenized and extracted in a 10 mL 20 mM phosphate buffer (pH 7.0) and centrifuged at 10,000 rpm for 15 min at 4 °C. A volume of 0.2 mL of the liquid supernatant was then mixed with 5 mL H_2_SO_4_ and 1.8 mL distilled H_2_O and reacted in a boiling water bath for 10 min. After cooling at room temperature, the absorbance was measured at 620 nm. To estimate the total soluble protein content, leaf samples were ground with liquid nitrogen, and 0.5 g samples were homogenized in a 1.5 mL ice-cold 50 mM phosphate buffer (pH 7.0) containing 1 mM ethylenediaminetetraacetic acid (EDTA), 0.05% (*v*/*v*) Triton X-100, and 1 mM polyvinylpyrrolidone (PVP). The extracts were then centrifuged at 13,000 rpm for 20 min at 4 °C, and the liquid supernatant was used to assay the soluble protein content.

### 4.5. Quantification of Endogenous Cytokinins and Auxins

Strawberry plants treated with 0.5 mg·L^−1^ TDZ were used for hormone content determination. The strawberry shoots kept at −80 °C refrigerator were taken out and ground into powder. In total, 50 mg samples were mixed with 10 μL 100 ng·mL^−1^ internal standard solution and 1 mL mixture of methanol, water, and formic acid (15:4:1, *v*/*v*/*v*). The samples were vortex mixed and centrifuged at 4 °C and 12,000 rpm for 5 min. The supernatant was transferred to a new centrifuge tube and evaporated to dryness by vacuum centrifugal evaporator. Then redissolved with 100 μL 80% methanol solution, and then a 0.22 μm filter membrane was used for filtration. The solution was then placed in sample vials for ultra-performance liquid chromatography (UPLC, ExionLC™ AD, AB Sciex Pte. Ltd., Singapore)—electrospray tandem mass spectrometry (QTRAP^®^ 6500+, AB Sciex Pte. Ltd., Singapore) [UPLC–ESI(+)–MS/MS] analysis.

A total of 2 μL of each sample was injected and analyzed with ACQUITY UPLC HSS T3 C18 1.8 µm column (Waters, Milford, MA, USA). The samples were analyzed in MRM mode and eluted using a 12 min gradient of 0.04% (*v/v*) acetic acid (A) and 0.04% (*v/v*) acetic acid in acetonitrile (B) at a flow rate of 0.35 mL·min^−1^. Gradient elution procedure: 95% A 5% B (0–1 min); 5% A 95% B (1–8 min); 5% A 95% B (8–9 min); 95% A 5% B (9–12 min). The column temperature was 40 °C. The temperature of Electrospray Ionization (ESI) was 550 °C. The mass spectrum voltage was 5500 V in positive ion mode and −4500 V in negative ion mode. The curtain gas was 35 psi. The standard curve was made using different CK, auxin, and their derivatives. A standard solution with 0.01 ng·mL^−1^ to 500 ng·mL^−1^ concentration was prepared, and the peak intensity data of each concentration standard were obtained. The standard curve of different substances is shown in Supplementary Table S1.

### 4.6. RNA Extraction and cDNA Library Construction

Strawberry plants treated with 0.5 mg·L^−1^ TDZ were used for transcriptome analysis. The strawberry plantlets were ground into powder in mortars with liquid nitrogen. Total RNA was extracted using the hexadecyltrimethylammonium bromide (CTAB) method [50], and DNase (Takara Bio, Beijing, China) was used to remove DNA interference. The concentration of RNA was measured by NanoDrop 2000 spectrophotometer (Thermo Scientific, Wilmington, NC, USA), and agarose gel electrophoresis was performed to check the integrity of the total RNA. RNA library preparation kit (New England BioLabs, Ipswich, MA, USA) was used to construct the library, the quality was assessed by Agilent 2100 Bioanalyzer, and the qualified cDNA library was used for RNA-seq and qRT-PCR.

### 4.7. RNA-Seq and DEGs Enrichment Analysis

All the strawberry samples were paired and sequenced using a HiSeq 2500 sequencer (Illumina, San Diego, CA, USA). In total, 6 GB of data were obtained for each sample. NGS QC Toolkit software v2.3 was used for quality control by removing low-quality bases and unknown N bases. High-quality clean reads were finally obtained. Clean reads were then assembled and mapped to the *Fragaria vesca* diploid strawberry genome from the GDR database (http://www.rosaceae.org). Differentially expressed genes (DEGs) between different samples were analyzed based on Cufflinks Software v2.2.1 according to the abundance of gene fragments. HTSeq-Count was used to obtain the number of gene reads in each sample. The function nbinomTest was used to calculate the *p*-value and fold change between samples. DEGs with *p*-value < 0.05 and |log2 (fold change)| > 1 were selected as candidate genes for analysis. Genes were annotated according to BLAST (https://blast.ncbi.nlm.nih.gov/Blast.cgi, accessed on 25 October 2024) results that were compared to sequences in several databases, including the Gene Ontology (GO), Kyoto Encyclopedia of Genes and Genomes (KEGG), Pfam, and NCBI databases.

### 4.8. Validation of Gene Expression Profiles by qRT-PCR

Nine candidate genes were selected to test the reliability of RNA-seq data by qRT-PCR. All the primers used for qRT-PCR analysis were designed by Primer Premier 5.0 (Table S2) without any interference of the conserved region and with an amplified product length of 200–500 bp. Actin 2 was selected as the housekeeping gene to normalize the results of the qPCR [51]. The qRT-PCR was performed using the SYBR® Premix Ex TaqTM II kit (Takara, Kyoto, Japan), as instructed. The cycling program was as follows: 95 °C for 3 min, followed by 40 cycles of 95 °C for 20 s, 58 °C for 20 s, and 72 °C for 30 s, followed by a melting curve analysis at 60–95 °C. The relative expression level of candidate genes was calculated using the comparative 2^−ΔΔ*C*T^ method [52]. In order to demonstrate the correlation between qRT-PCR and RNA-Seq analysis, the results were presented as log_2_ (TDZ/Control).

### 4.9. Data Collection and Analysis

Statistical analysis was carried out using the SPSS-23 software. Experimental results of plant growth were subjected to an analysis of one-way ANOVA (*p* ≤ 0.05) and Duncan’s multiple range test (*p* ≤ 0.05) since they are single-factor experiments. The dynamic data of cytokinins and auxins contents were subjected to an analysis of two-way ANOVA followed by Duncan’s multiple range test at *p* ≤ 0.05. Graphing was performed using OriginPro software (version 9.0).

## Data Availability

The raw data supporting the conclusions of this article will be made available by the authors on request.

## Supplementary Materials

The following supporting information can be downloaded at: https://www.mdpi.com/article/10.3390/ijms26094060/s1.
