# Supplementary material for: Thidiazuron Enhances Strawberry Shoot Multiplication by Regulating Hormone Signal Transduction Pathways
Source: Int J Mol Sci. 2025 Apr 25;26(9):4060. doi: 10.3390/ijms26094060 (PMC12071388; doi:10.3390/ijms26094060)
Supplement: Supplementary file 1 [file ijms-26-04060-s001.zip › ijms-3529745-supplementary.pdf]

Table S1 Linear equations and correlation coefficients of the standard curves of tested substances

Index	Compounds	Class	RT	Equation	r	Weighting	LLOQ	ULOQ
TRP	L-tryptophan	Auxin	3.15	y=0.00661x+0.00601	0.99922	1/x	2	10000
ICAld	Indole-3-carboxaldehyde	Auxin	5.02	y=0.04766x+0.03632	0.99808	1/x	0.1	500
IAA	Indole-3-aceticacid	Auxin	5.16	y=0.01421x+5.84948e^{-4}	0.99987	1/x	0.2	500
Indole	Indole	Auxin	N/A	y=0.00480x+0.00318	0.99917	1/x	0.5	500
tZRMP	9-Ribosyl-trans-zeatin5'-monophosphate	CK	1.74	y=0.02928x-5.28895e^{-4}	0.99495	1/x	2	500
tZ	trans-Zeatin	CK	2.34	y=0.17074x+0.00312	0.99961	1/x	0.1	500
DHZROG	Dihydrozeatin-O-glucoside riboside	CK	3.58	y=0.39214x+0.00314	0.99975	1/x	0.1	500
cZROG	cis-Zeatin-O-glucosideribo-side	CK	3.61	y=0.02371x+1.76233e^{-4}	0.99665	1/x	0.1	500
tZR	trans-Zeatinriboside	CK	3.68	y=0.15341x+0.00186	0.9957	1/x^2	0.1	500
IP	N6-isopentenyladenine	CK	4.24	y=0.07011x+0.00776	0.99672	1/x	0.1	500

Table S2. The details of primers used for RT-qPCR

Gene id	Primer	Primer sequence (5′ → 3′)	Annealing Temperature (°C)	Amplicon size (bp)
FvH4_1g07610	CKX2-F	TTCGGCTCTCGGAGGTTTAGGC	64	214
	CKX2-R	AGGACCCTGCGGCATTAGAACA		
FvH4_1g07620	CKX3-F	GGCTCGTAATGGAGTCGTGGTC	62	216
	CKX3-R	CCTCCGACGGTCAGGTACAAGT		
FvH4_2g39230	CKX5-F	TTGTCAGTCGGCGGTACACTCT	63	220
	CKX5-R	GGGCTGGTTCAAGGGCAATTCT		
FvH4_4g14440	IPT5-F	GGAGCAAGTGGAACTGGAGCAT	62	250
	IPT5-R	GCCGACATGGACACCGTTAGAG		
FvH4_6g17020	UGT73C1-F	CGCAGACTTATGCTTGCCTTGG	62	224
	UGT73C1-R	GCACTGGTCCTGGTATCTGAGC		
FvH4_3g01870	AHP-F	TCAAGATGAGAGCACCCCAG	61	234
	AHP-R	CCTTCAAAATGTTCTCTTCTTC		
FvH4_6g45180	CYP-F	GTGGAGAGGCTGCTGATGGAGA	63	222
	CYP-R	GACGGTCCAAGTGAGCAAGAGG		
FvH4_1g16680	CYCD3-F	TGAGGGAGAAGAAGAAGCAG	63	225
	CYCD3-R	CTAGCCAACGTTAAAGACCC		
FvH4_4g24870	ARR11-F	ATGAAGCATTCTGATTATCC	60	254
	ARR11-R	ACTTGTGAACTTCTGGTCTT		

Table S3. Quality assessment of RNA-Seq data.

Sample Name	Raw Reads （M）	Raw Base （G）	Clean Reads （M）	Clean Base （%）	Vaild Base （%）	Q30 （%）	GC content （%）
CK-1	49.93	7.22	47.62	6.88	95.37	94.69	47.08
CK-2	51.11	7.40	48.74	7.06	95.37	94.66	46.98
CK-3	50.99	7.39	48.75	7.06	95.61	94.67	47.13
TDZ-1	50.76	7.33	48.30	6.97	95.17	94.71	46.98
TDZ-2	50.95	7.30	47.92	6.87	94.06	94.64	46.81
TDZ-3	47.05	6.84	45.16	6.56	95.99	94.39	47.07
